# How coping can hide larger systems problems: the routine immunisation supply chain in Bihar, India

**DOI:** 10.1136/bmjgh-2019-001609

**Published:** 2019-09-05

**Authors:** Bruce Y Lee, Patrick T Wedlock, Elizabeth A Mitgang, Sarah N Cox, Leila A Haidari, Manoja K Das, Srihari Dutta, Bhrigu Kapuria, Shawn T Brown

**Affiliations:** 1 Global Obesity Prevention Center (GOPC), Johns Hopkins University, Baltimore, Maryland, USA; 2 Public Health Informatics, Computational, and Operations Research (PHICOR), Baltimore, Maryland and New York City, New York, USA; 3 HERMES Logistics Team, Pittsburgh, Pennsylvania and Baltimore, Maryland, USA; 4 INCLEN Trust International, New Delhi, India; 5 UNICEF India, New Delhi, India; 6 UNICEF Regional Office for South Asia, Kathmandu, Nepal; 7 McGill Center for Integrative Neuroscience, McGill University, Montreal, Quebec, Canada

**Keywords:** health systems, vaccines

## Abstract

**Introduction:**

Coping occurs when health system personnel must make additional, often undocumented efforts to compensate for existing system and management deficiencies. While such efforts may be done with good intentions, few studies evaluate the broader impact of coping.

**Methods:**

We developed a computational simulation model of Bihar, India’s routine immunisation supply chain where coping (ie, making additional vaccine shipments above stated policy) occurs. We simulated the impact of coping by allowing extra trips to occur as needed up to one time per day and then limiting coping to two times per week and three times per month before completely eliminating coping.

**Results:**

Coping as needed resulted in 3754 extra vaccine shipments over stated policy resulting in 56% total vaccine availability and INR 2.52 logistics cost per dose administered. Limiting vaccine shipments to two times per week reduced shipments by 1224 trips, resulting in a 7% vaccine availability decrease to 49% and an 8% logistics cost per dose administered increase to INR 2.73. Limiting shipments to three times per month reduced vaccine shipments by 2635 trips, which decreased vaccine availability by 19% to 37% and increased logistics costs per dose administered by 34% to INR 3.38. Completely eliminating coping further reduced shipments by 1119 trips, decreasing total vaccine availability an additional 24% to 13% and increasing logistics cost per dose administered by 169% to INR 9.08.

**Conclusion:**

Our results show how coping can hide major system design deficiencies and how restricting coping can improve problem diagnosis and potentially lead to enhanced system design.

**Key question**

**What is already known?**

- Previous studies on coping mechanisms in health systems have focused largely on determining how and how much of these behaviours occur in clinical settings, particularly for nurses and physicians.
- Multiple studies have demonstrated how healthcare workers often use coping mechanisms, or ‘workarounds’, when required to adapt to a new technology, policy or obstacle in clinical settings.

**What are the new findings?**

- Our research demonstrates and quantifies how persistent coping behaviours may mask deeper systems problems, such as cold chain constraints and inadequate policies.
- Furthermore, our study quantifies the resulting impact when coping mechanisms are removed from the system.

**What do the new findings imply?**

- Those who run health systems, such as immunisation systems, need to more closely detect, monitor and mitigate the need for coping by exposing and properly addressing problems.
- As our study shows, continuing to rely on coping has substantial risks. When healthcare personnel are no longer able to continue coping, the system can fall apart very quickly.
- Instituting anti-coping measures, such as policies to establish a culture where coping is discouraged, and measuring the amount of coping in a system, can improve system function.

## Introduction

Coping occurs when personnel working in a health system make additional, often undocumented efforts to compensate for deficiencies in the existing design or management of the system. Coping may occur in a wide variety of situations. One example is when circumstances change, but an existing system has not changed to match or compensate for the new situation. Another example is when a new policy or technology is introduced without considering the broader resulting impact on those inside and outside the system such as health professionals working extra hours to complete work or going beyond established protocols to ensure that a task is completed.^1–3^

In certain situations, coping can be a positive phenomenon. They can emerge from good intentions and help the system deliver the intended primary outcomes. As Abimbola and Topp have indicated,^4^ coping can be part of a system’s resilience, the ability of a system to accommodate unexpected situations. The baseline design and operation of a system cannot possibly account for every situation. Therefore, a system’s resilience depends on whether parts of the system can adapt, especially when changes in circumstances are temporary.

However, there is a difference between having a resilient system with the ability to cope to changes versus a deficient system in which coping hides serious deficiencies. When a system as it is designed cannot handle even prevailing conditions, coping can mask the need for significant changes and leave the system unstable. Coping can actually decrease a system’s resilience by overstretching parts of the system, especially when such coping goes undocumented and depends disproportionately on certain system parts.

Immunisation systems serve as good exemplars to better understand the potential impact of coping. As our previous studies demonstrated, the design of most low-income and middle-income countries’ routine immunisation systems took place around four decades ago.^5–8^ Despite changes in external circumstances (eg, substantial population growth) and the introduction of new technology (eg, vaccine introductions), there have been relatively few re-design efforts until recently.^5 9 10^ This raises the possibility that significant coping could be occurring.

Since immunisation is crucial to protecting populations against potential life-threatening or life-altering diseases, the consequences of coping masking inadequate immunisation systems can be substantial. They can also be complex and difficult to follow unaided as immunisation supply chains are complex.^11 12^ Therefore, to assess the broader impact of varying degrees of coping behaviours, we developed a computational simulation model of the routine immunisation supply chain of Bihar (a state in India), where coping (in this case, making additional shipments of vaccines above stated policy) is known to occur.

## Methods

### HERMES

As described in previous publications,^7 9 10 13–16^ Highly Extensible Resource for Modelling Event-Driven Supply Chains (HERMES) is a software platform developed by the HERMES logistics team that allows users to generate detailed discrete event simulation models of any vaccine supply chain. Each supply chain model contains a virtual representation of all storage facilities and devices (including buildings, refrigerators and freezers), vehicles and routes (including vehicle types, travel frequency and travel distance), human resources (including logisticians, drivers and vaccinators), vaccines, supply chain policies and associated costs for each component.

At each virtual immunisation location, virtual people arrive each session when they are ready to receive a particular vaccine or set of vaccines. If the correct vaccine is available at the immunisation location, then the person is successfully immunised. If the vaccine is not available, then the person counts as a missed vaccination opportunity. Unused doses in open vaccine vials that must be discarded accrue as open vial wastage.

#### HERMES-generated model of Bihar, India’s Universal Immunisation Program supply chain

**Figure 1:**
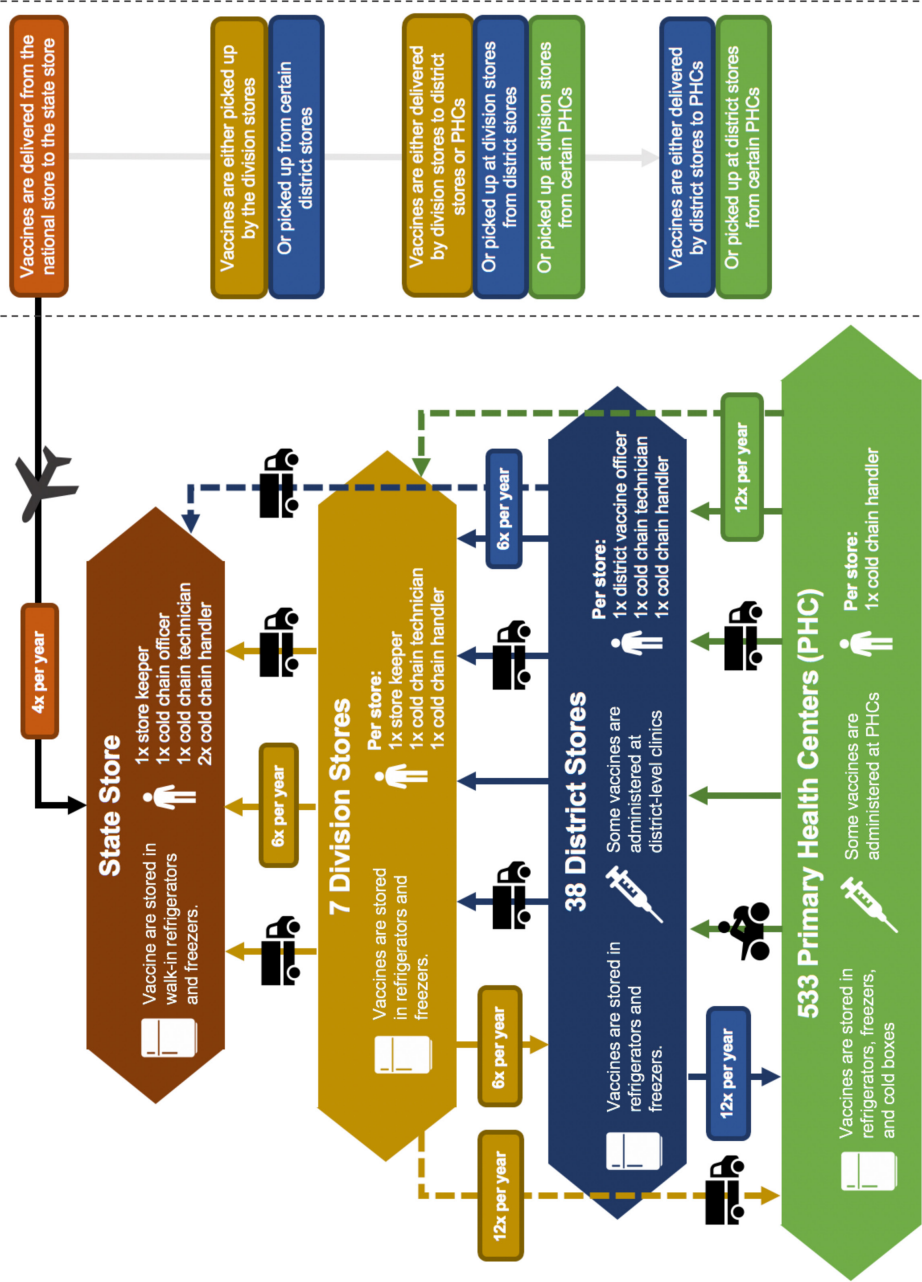
Diagram of the universal immunisation programme supply chain in Bihar, India.

Our team developed a HERMES-generated model of Bihar, India’s immunisation supply chain (figure 1) comprised of four main levels: one State store in Patna, seven Division stores, 11 (of 38 total) District stores and 161 (of 533 total) primary health centre (PHC) stores. The model includes characteristics of the 2018 Universal Immunisation Programme (UIP) vaccines in India.^17^ The HERMES Logistics Team and Public Health Informatics, Computational, and Operations Research team developed the model with support from the Public Health Foundation of India and the INCLEN Trust International, a New Delhi-based global health non-profit research organisation, who collected data on cold chain equipment, supply chain structure, population and associated costs for this model in 2013. INCLEN utilised a data collection tool adapted from effective vaccine management, vaccine management assessment tool, cold chain equipment management, HERMES and national guidelines and modified with input from members of the multi-disciplinary technical expert group, HERMES Logistics Team, and Immunization Technical Support Unit (ITSU).

The model includes data from the State store in Patna and all seven Division stores. Within these seven Divisions, the research team collected data from 13 Districts including Aurangabad, Banka, Buxar, Gopalganj, Jamui, Kaimur, Katihar, Khagaria, Madhubani, Muzaffarpur, Nawada, Supaul and West Champaran. Within each of these districts, the research team visited all the PHC vaccine stores (n=161) and documented the cold chain device details, cold chain space, vaccine stock, vaccine logistics and transportation network, population served, energy and maintenance mechanisms, and associated outreach locations.

For the remaining Districts and PHCs in Bihar not captured during the data collection phase, our team assigned the population of each location to ‘surrogate’ stores modelled at the Division and State level. This means that both the State and Division stores in the model receive vaccines to cover the population for all of Bihar. The surrogate populations allow the model to identify and quantify any storage or transport constraints at the State and Division levels. However, once vaccines leave the Division store to the surrogate population, these vaccines are not counted in our supply chain metrics like vaccine availability or logistics costs.

The median population served at the 161 represented PHC stores in the 13 Districts totals 229 136 (range: 21 000–4 62 414). The District stores serve a median population of 2 495 377 (1 666 886–4 587 945). To determine the number of newborns requiring vaccines at each immunisation location, we applied a crude birth rate of 2.77% to each location’s total population served.^18^ The 2011–2012 Bihar population served across all Divisions, as reported by INCLEN, was 103 804 637 resulting in an annual birth cohort of 2 875 388. We then extrapolated this population to represent the 2018 population by applying a population growth rate of 2.1%^18^ (assuming a constant growth rate from previous years), yielding a total population of 115 166 679 and an annual birth cohort of 3 190 117. We applied an infant mortality rate of 4.4% to each newborn population to determine the population of infants under 1 year of age and an under-five mortality rate of 5.9% to each newborn cohort to determine the population of children requiring booster immunisations.^18^

#### Introducing and removing coping mechanisms from the supply chain

According to national guidelines at the time of the study, vaccine shipments are limited to six times per year between the State and Division stores and between the Division and District stores, and 12 times per year between the District/Division stores and the PHCs. Based on empirical data from INCLEN and ITSU, many shipments in Bihar happen more frequently than per policy, some more than three times per month. In order to capture the effects of coping in the form of increased transport frequencies, we modelled three coping scenarios based on country input and a fourth scenario with coping mechanisms completely removed.

- *Extensive coping*: Allowing shipments to occur as needed up to one time per day across all routes.
- *Moderate coping*: Allowing shipments to occur up to two times per week across all routes.
- *Low coping*: Allowing shipments to occur up to three times per month across all routes.
- *No coping*: Restricting shipments to follow stated policy.

Under extensive coping conditions, we included three additional experiments varying the likelihood of shipping delays within the system. These included: 10-day shipping delays occurring once every 10 trips on routes to retrieve vaccines from the State store; 10-day shipping delays occurring once every 10 trips on all routes between the State store, Division stores and District stores, and 10-day shipping delays occurring once every 10 trips across all routes. Additional scenarios increased the population by 10% to 20%, which is what could occur over the next decade or two based on current growth rates. Another set of scenarios simulated the impact of introducing a new vaccine, the human papillomavirus (HPV) vaccine, to the UIP.

For the no coping scenario, we included two additional experiments: varying the storage capacity of vehicles and refrigerators/freezers throughout the supply chain to demonstrate the relative impact of these compared with coping. Specifically, we tested the impact of doubling vehicle capacity across all routes and^2^ the impact of doubling the storage capacity across all locations.

For each scenario, HERMES models generate a number of outputs, including vaccine availability (ie, the percentage of clients arriving at an immunisation location who are successfully vaccinated), storage and transport capacity utilisation (eg, the percentage of available space used each day), number of trips between any two points in the network and number of stockouts (ie, the number of times a location runs out of a particular vaccine). In addition, the model captures the total logistics costs and vaccine procurement costs per year, per dose administered and per fully immunised child.

#### Patient and public involvement

This study did not include any identifiable data on human subjects and therefore would be exempt from an institutional review board review.

## Results

**Figure 2:**
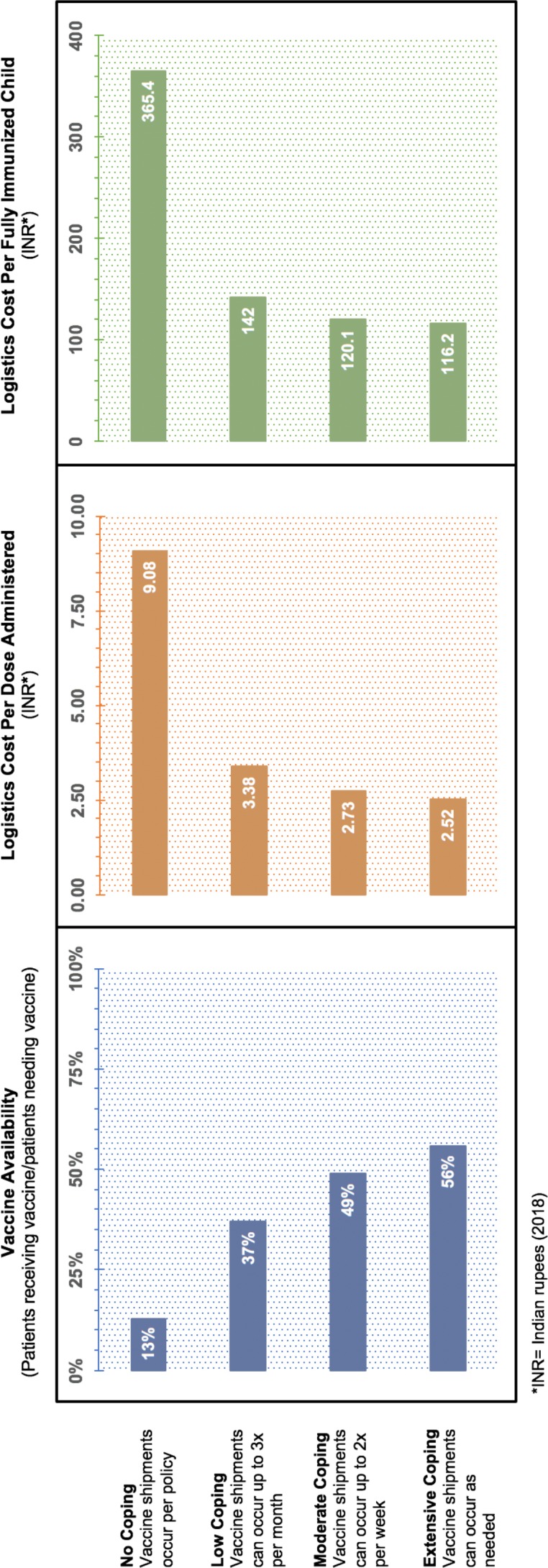
Change in key supply chain metrics under different coping scenarios.

**Figure 3:**
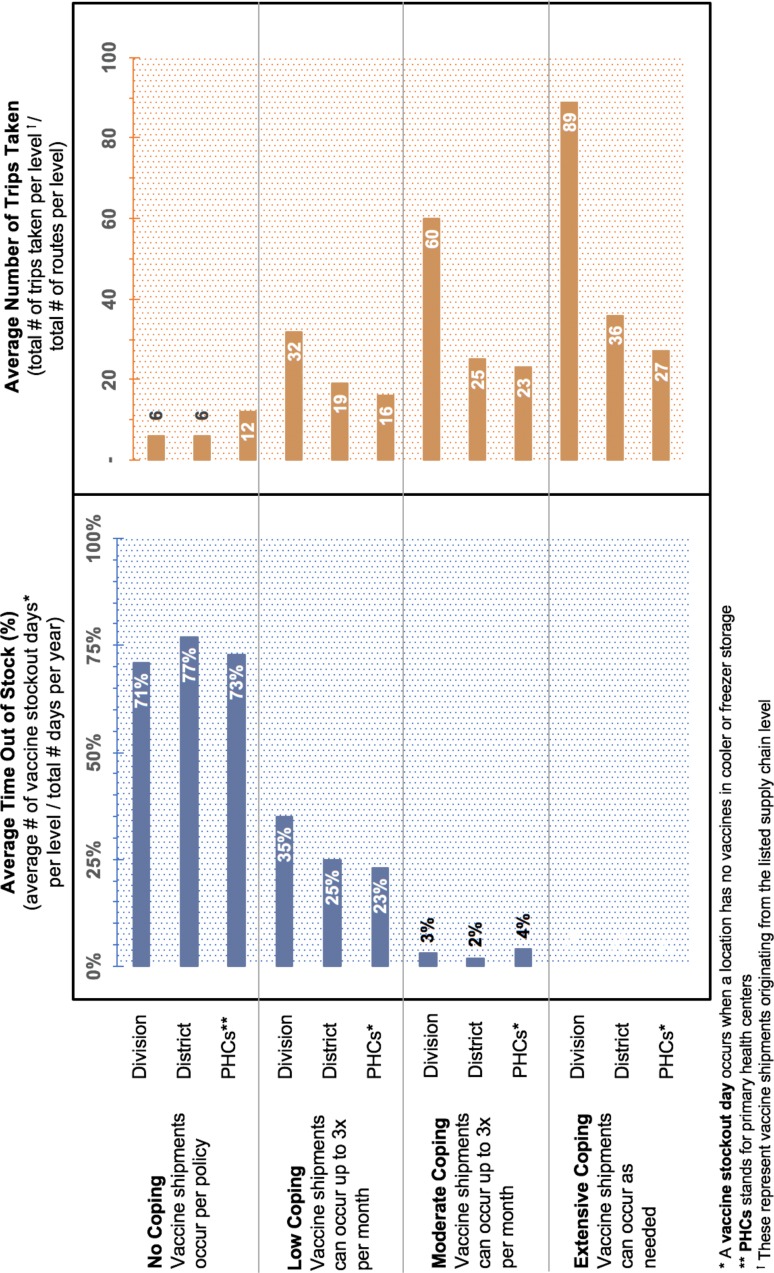
Average time out of stock and trips taken under different coping scenarios.

Each simulation runs at a daily resolution over 1 year; results reflect the average of 23 runs. Table 1 provides results for total vaccine availability, shipments made and logistics costs for each scenario. Figures 2 and 3 show the change in key supply chain metrics under different coping scenarios. All costs are reported in 2018 Indian rupees (INR).

**Table 1:** Supply chain metrics across four coping scenarios in Bihar’s vaccine supply chain

Coping scenario	Total vaccine availability	Total vaccine shipments made	Total logistics costs (2018 INR)	Logistics cost per dose administered (2018 INR)
Extensive*	56%	5746	41 843 849	2.52
Moderate†	49%	4522	39 800 907	2.73
Low‡	37%	3111	37 337 839	3.38
None§	13%	1992	35 152 981	9.08

*Extensive coping means vaccine shipments can be made up to one time per day.

†Moderate coping means vaccine shipments can be made up to two times per week.

‡No coping means vaccine shipments must be made according to policy.

§Low coping means vaccine shipments can be made up to three times per month.

### Any degree of coping is allowed

Under extensive coping conditions (ie, vaccine shipments can be made as frequently as needed, up to one time per day), each modelled route in the Bihar vaccine supply chain makes at least one additional trip above the stated policy.

On the routes to retrieve vaccines from the State store (n=10, including seven originating from Division stores and three originating from District stores), the average number of extra trips made annually per route is 83 (range: 23–205). This represents a 1375% increase in the average number of trips made per route compared to when the supply chain follows transport policy. Shipments occurring at this level of the supply chain are particularly constrained as the average transport capacity per vehicle (300 L, range: 160–3000 L) is far less than would be needed to meet the expected bimonthly demand for vaccines (958–7 098 L) when following the current transport policy.

On the routes to retrieve vaccines from the Division stores (n=34, including eight originating from the District stores and 26 originating from PHCs), the average number of extra trips made annually per route between these locations is 25 (range: 4–205), representing a 245% increase in the average number of trips made per route compared with per policy. Three routes (departing from Gopalganj, West Champaran and Madhubani, respectively) are particularly overutilised, making between 55 and 205 additional trips per year. Increased utilisation on these routes is due to highly constrained transport and storage capacity, as well as low vaccine stocks at the Division stores.

On the routes to retrieve vaccines from the District stores (n=135, all originating from PHCs), the average number of extra trips made annually per route between these locations is 15 (range: 1–191), representing a 126% increase per route compared with per policy. While coping still occurs at this level of the supply chain, the extent of the coping is much smaller compared with the higher levels. This is due to the fact that transport capacity (~80 L on average) and the frequency of trips allowed (monthly) are often satisfactory to meet the demand for vaccines. At this level, coping occurs most often on routes using three-wheeler vehicles with a transport capacity of only 10 L, or where vaccine stocks at the District stores are regularly depleted.

Even when vehicles are allowed to travel up to one time per day, the average peak transport utilisation—that is, the maximum percentage of available transport capacity needed to complete any shipment—is 228%, across all routes in the supply chain. A peak transport capacity utilisation above 100% indicates that a vehicle does not have enough storage space to adequately carry the entire demanded shipment. In this scenario, on average across all routes, a vehicle would need 2.28 times the available cold storage space to deliver the entire shipment. The highest average peak transport utilisation occurred in vehicles retrieving vaccines from the State store (1309%, range: 6%–2111%).

The increase in shipments made across each level of the supply chain results in a total vaccine availability of 56% (table 1), which is a 43% increase compared with the system when the transport policy is enforced. However, these trips also result in increased transport costs, which includes an increase in annual fuel costs (+INR 4.3 million), vehicle depreciation costs (+INR 1.7 million) and vehicle maintenance costs (+INR 0.6 million). The total logistics cost per dose administered, a composite metric of both vaccine availability and total logistics costs, is INR 2.52 per dose administered, representing a 72% decrease in price per dose compared with per policy.

When 10-day shipping delays occurred at a rate of once every 10 trips between the top two levels, the number of shipments over stated policy decreased by 307 from 3754 to 3447—an 8% decrease—resulting in a 4% decrease in vaccine availability from 56% to 52%. When 10-day shipping delays occurred at a rate of once every 10 trips between the top three levels—State, Divisions and District routes—the number of shipments over policy decreased by 650 from 3754 to 3104—a 17% decrease—resulting in a 7% decrease in vaccine availability from 56% to 49%. And when 10-day shipping delays occurred at a rate of once every 10 trips across all routes, the number of shipments over stated policy decreased by 1287 from 3754 to 2467—a 34% decrease—resulting in an 11% decrease in vaccine availability from 56% to 45%.

When the population increased by 10%, the number of shipments over policy increased by 268 trips per year with more routes increasing shipment frequency up to one time per day. Yet, vaccine availability still decreased by 4% (from 56% to 52%). When the population increased by 20%, the number of shipments over policy increased by an additional 184 trips but still resulted in further reduction of vaccine availability to 50%. Introducing the HPV vaccine into the UIP resulted in increases in total vaccine shipments by 252 trips per year but nonetheless led to a decrease in vaccine availability from 56% to 50%.

### Moderate degree of coping is allowed

Under moderate coping conditions (ie, vaccine shipments can be made as needed up to two times per week), 167 of the 179 modelled routes (93%) make at least one additional shipment above stated policy.

On the routes to retrieve vaccines from the State store, 100% of vehicles take at least one additional trip above policy. The average number of extra trips made annually per route between these levels is 54 (range: 17–65), representing a 900% increase in the average number of trips made per route. On the routes to retrieve vaccines from the Division stores, 91% of vehicles take at least one additional trip above stated policy, and all routes originating from the District stores (n=8) take at least one additional trip. The average number of extra trips made annually per route between these locations is 14 (range: 1–57), representing a 138% increase in the average number of trips made per route.

On the routes to retrieve vaccines from the District stores, 93% of vehicles take at least one additional trip above stated policy when moderate coping is allowed to occur. The average number of extra trips made annually per route between these locations is 11 (range: 0–59), representing a 91% increase in the average number of trips made per route.

The increase in shipments made across each level of the supply chain results in a total vaccine availability of 49% under moderate coping conditions, an increase in 36% compared with per policy (table 1). However, these trips also incur increased transport costs compared to when no coping is allowed. Under moderate coping conditions, annual fuel costs increase by INR 3.0 million, vehicle depreciation costs increase by INR 1.2 million and vehicle maintenance costs increase by INR 0.4 million. The logistics cost per dose administered is a ratio of total logistics costs divided by the total number of doses administered. The total logistics cost per dose administered is INR 2.73 under moderate coping, representing a 70% decrease in cost per dose compared with per policy. Conversely, although total logistics costs are 5% less than when shipments occur as frequently as needed, the total number of doses administered decreases by 12% resulting in a higher total logistics cost per dose administered compared with when shipments occur as needed.

### Low degree of coping is allowed

Under low coping conditions (ie, vaccine shipments can be made as needed up to three times per month), 121 of the 179 modelled routes (68%) make at least one additional shipment above stated policy.

On the routes to retrieve vaccines from the State store, 100% of vehicles take at least one additional trip above policy. The average number of extra trips made annually per route between these levels is 26 (range: 13–29), representing a 432% increase in the average number of trips made per route. Nine of these ten routes have transport storage capacities of 300 L or less per shipment, which is far short of the capacity needed to meet demand. For the one route that has adequate transport capacity (departing from Khagaria with 3000 L capacity), the refrigerator capacity at the District store is not adequate to hold that quantity of vaccines. As such, each of these routes must make at least one additional trip over stated policy.

On the routes to retrieve vaccines from the Division stores, 70% of vehicles take at least one additional trip above policy, and all routes originating from the District stores (n=8) take at least one additional trip. The average number of extra trips made annually per route between these locations is 8 (range: 0–26), representing a 77% increase in the average number of trips made per route.

On the routes to retrieve vaccines from the District stores, 55% of vehicles take at least one additional trip above stated policy when low coping is allowed to occur. The average number of extra trips made annually per route between these locations is 4 (range: 0–24), representing a 34% increase in the average number of trips made per route. Unlike the system with extensive coping conditions, multiple routes originating from the lowest level of the supply chain (ie, PHCs) do not take additional trips. This is primarily due to stockouts occurring at the stocking locations.

When shipments are allowed to occur up to three times per month, the average peak transport utilisation is 229% under both moderate and low coping conditions. The highest average peak transport utilisation occurred in vehicles retrieving vaccines from the State level (1308%, range: 6%–2111%). For trips taken to retrieve vaccines from the Division stores, average peak transport utilisation was 176% (range: 15%–990%), and for trips taken to retrieve vaccines from the District stores, average peak transport utilisation was 162% (range: 19%–1213%).

The increase in shipments made across each level of the supply chain results in a total vaccine availability of 37% under low coping conditions, an increase in 24% compared with per policy (table 1). However, these trips also incur an increase in transport costs compared with when no coping is allowed. Under low coping conditions, annual fuel costs increase by INR 1.4 million, vehicle depreciation costs increase by INR 0.6 million and vehicle maintenance costs increase by INR 0.2 million. The total logistics cost per dose administered is INR 3.38 under low coping, representing a 63% decrease in cost per dose compared with per policy. Conversely, although total logistics costs are 11% less than when shipments occur as frequently as needed, the total number of doses administered decreases by 33%, resulting in a higher total logistics cost per dose administered compared with when shipments occur as frequently as needed.

### No coping is allowed

When vaccine shipments are required to occur according to the set policy, an average of six trips are made to retrieve vaccines from the State store over the course of a year. On the routes to retrieve vaccines from the Division stores, the average number of trips is 11, and on the routes to retrieve vaccines from the District stores, the average number of trips is 12. Under this policy, only 89 of the 179 routes modelled are able to deliver any vaccines. The remaining routes (50%) cannot retrieve any vaccines from the level above, due to vaccine stockouts, resulting in a 75% decrease in the total number of vaccines delivered to the PHCs compared with when extensive coping is allowed (table 1). As such, removing all of the transport coping mechanisms from the supply chain results in a vaccine availability of just 13%. While recurring transport costs are lower compared with the coping scenarios described above, the total number of doses administered decrease between 65% and 77% resulting in a logistics cost per dose administered of INR 9.08.

Doubling the transport capacity of each vehicle in the supply chain under no coping conditions improves vaccine availability from 13% to 20%. While no additional shipments can be made above stated policy, the system is able to deliver 27 267 additional litres of vaccine. However, vaccine availability under these conditions (20%) is substantially less than when any degree of coping occurs (37%–56%).

Doubling the storage capacity at each of the locations in the supply chain improves vaccine availability from 13% to 25%. Under these conditions, the system is able to deliver 51 286 additional litres of vaccine compared with baseline. Again, however, vaccine availability under these conditions (25%) is substantially less than when any degree of coping occurs (37%–56%).

## Discussion

Our study demonstrates how existing coping mechanisms can mask significant deficiencies in the design of a health system. Immunisation supply chains are complex systems with interconnected and interrelated components and processes that may appear to be functioning correctly when coping is occurring. For example, the amount of cold chain capacity being utilised may seem adequate if personnel are making more frequent shipments that relieve cold chain constraints. By demonstrating and removing these coping behaviours in Bihar’s immunisation supply chain, our study identifies a number of design, structure and policy flaws that need to be addressed, such as an insufficient shipment policy that does not match system capacity, inefficient use of available resources and constrained vaccine transport and storage space. Fixing such flaws is not necessarily an overwhelming and cost prohibitive task. For example, published work in Benin^9 15 19^ has shown that re-designing the immunisation supply chain can actually end up saving money immediately and over time due to gains in efficiency.

There has been a dearth of studies showing how persistent coping behaviours may hide systems problems and quantifying the resulting impact. Available coping-related studies mostly focus on determining how much coping or ‘workarounds’ occur after a new policy or technology was introduced to a hospital or clinic.^1–3 20–31^ In other words, they focus on what healthcare workers may do to avoid adopting a new situation like healthcare personnel reverting to using paper-based methods when electronic health record technology is introduced or what personnel do besides using new patient identification barcodes because the barcodes are unreliable. However, few studies examine longer term and wider implications of chronic coping. This could have substantial implications as anecdotal evidence suggests that coping is going on frequently throughout many different health systems worldwide. This includes stories of healthcare personnel spending non-working hours completing their work,^1^ taking on tasks for which they are untrained or unprepared,^3^ and circumventing protocols to administer medications during shortages.^32^

As our study results suggest, it is important to understand the broader impact of coping since such behaviours can actually bring substantial risks. While short-term coping behaviours to overcome temporary situations may be a sign of resilient health system, the existence of chronic coping behaviours to accommodate prevailing conditions may be an indication that deeper systemic issues exist and need to be addressed.^4 28 33^ Therefore, when assessing coping, one has to distinguish between temporary versus chronic coping and determine the threshold at which coping has occurred for too long. Additionally, one should determine what parts of the system are using coping and how this relates to the rest of the system.

The Bihar routine immunisation supply chain is an example of a system in which one set of personnel, those at the outermost level of the supply chain, are coping to support the deficiencies of the system. Thus, they bear a disproportionate burden in supporting the system, leaving them overstretched and in a potentially very unstable situation. If circumstances were to further change or these personnel are no longer able to cope, the entire system could break down very quickly. In truly resilient systems, responsibility is more distributed and redundant. Relying so heavily on the outermost part of supply chain is fraught with problems. For example, it is more difficult to monitor and manage this part of the supply chain. Personnel and practices may be more variable. Once vaccines have reached this level, changes (eg, re-routing vaccines to and from other regions) may be more difficult to implement. Moreover, personnel at this level often have many other responsibilities and may not have the bandwidth to cope. It is unclear what other responsibilities are being forsaken (eg, healthcare workers not tending to patients' other concerns) due to having to cope.

All of this emphasises the need to more closely detect, monitor and mitigate the need for coping by exposing and properly addressing problems. Doing so requires a multi-pronged approach:

1. One prong is to establish a culture that encourages personnel to speak out when coping is occurring and helps identify and discourage ‘unhealthy’ coping. Unhealthy coping is any situation in which coping masks existing design issues or creates a risky situation. Such a culture should not suppress complaints or should not apply pressure to personnel to continue coping. This can be accomplished in part by openly discussing and assessing the risks of coping and maintaining open communication that allow personnel to reveal their coping behaviours without fear of reprisal.^29 34 35^
2. A second prong is to establish ways to monitor the type and amount of coping occurring. This includes establishing appropriate metrics to follow that do not just monitor the ultimate outcomes (eg, vaccine coverage) but also the intermediate steps (eg, number of trips) to achieve the outcomes and understanding the pros and cons of each metric. There should be ways in which these metrics can be adequately measured that do not impose too much burden on particular personnel. Such metrics should be not only quantitative (eg, logs of trips) but also qualitative (eg, asking staff about how they feel about their work and the accompanying challenges) to be able to capture the breadth of coping that is resulting and the drivers.^36–38^
3. A third prong is to establish policies and procedures that prevent unhealthy coping. Examples include maintaining clear standard operating procedures and limiting excursions from such procedures (eg, limiting access to an information system to working hours). This may be necessary because personnel who do not have a systems-wide view may continue to cope from good intentions without realising the broader impact. Moreover, unhealthy coping may set a precedent for others.
4. A fourth prong is to use systems methods to better diagnose coping situations and determine their impact. As we have previously indicated,^8 11 12 39^ without assistance, humans can struggle to understand the reverberating effects of a change throughout a complex system. Systems mapping and modelling can help elucidate and quantify such effects and identify ways to address resulting problems.
5. A final prong is to continually evaluate the design of systems and identify ways to re-design them if needed. Since circumstances change (eg, populations grow and diseases and technology evolve), truly resilient systems must also be able to change accordingly. The measure of a system's resilience is not just its ability to deliver desired outcomes but how it does so. Systems methods can help identify the appropriate system designs, as computational modelling has helped guide the re-design of immunisation systems in various countries.^9 11 15^ Re-designs may include changing the structure, the workflows, the type of personnel and the technology used in a system.^38 40 41^

Computational models, while attempting to represent real life, ultimately simplify complex components and processes. The model developed for this study aims to represent the complex system of infrastructural components, human resources and policies that comprise Bihar’s vaccine supply chain. However, based on the inherent nature of computational modelling, not all factors influencing vaccine delivery can be captured. To overcome data limitations, we modelled unsampled districts as a surrogate store with a surrogate population, which allows the model to show what bottlenecks may exist at the state or division levels. Otherwise, omitting the surrogate population could give the appearance of excess capacity at the top levels of the system.

## Conclusion

Our results show how coping can hide major deficiencies in the design of a system and how restricting coping can lead to better diagnosis of these problems and potentially lead to improved system design. While coping, that is, making improvisational, and often undocumented efforts to compensate for deficiencies in existing systems and management, can be well-meaning, it can in the long run do more harm than good. This suggests that decision-makers may want to install policies and interventions to prevent, monitor and reduce coping so that systems problems can be exposed and more properly addressed.
